# Interactions of Oxygen Vacancies with Photoinduced {Hole/Electron} Pairs in SrTiO_3–*x*_: Their Key Role in Photocatalytic H_2_ Production

**DOI:** 10.1021/acs.jpcc.5c03464

**Published:** 2025-08-15

**Authors:** Areti Zindrou, Loukas Belles, Yiannis Deligiannakis

**Affiliations:** Laboratory of Physical Chemistry of Materials & Environment, Department of Physics, 37796 University of Ioannina, GR-45110 Ioannina, Greece

## Abstract

The present work elucidates the role of lattice oxygen vacancies (V_o_s) in SrTiO_3_ (STO) nanoparticles on the spin dynamics of photogenerated charge carriers (electrons/holes, e^–^/h^+^) and on the photocatalytic hydrogen (H_2_) evolution from H_2_O. V_o_-enriched STO materials (V_o_-STO) were synthesized via anoxic flame spray pyrolysis (A-FSP) technology that allowed production of a library of SrTiO_3–*x*_ nanomaterials with controlled V_o_ concentrations. The optimal V_o_-STO materials exhibited a 200% increase in photocatalytic H_2_ production rates compared with pristine STO. A combined study using electron paramagnetic resonance spectroscopy and photoelectrochemistry reveals that monomeric oxygen vacancies (type-B V_o_s) are the key factors to boost photoinduced charge separation via suppression of the e^–^/h^+^ recombination. Mott–Schottky and electrochemical impedance spectroscopy show that increased surface V_o_ population results in a slight upshift of flat band potential (*E*_fb_) and decreases the interfacial charge-transfer resistance, hence enhancing photocatalytic activity. Furthermore, open-circuit potential decay measurements reveal longer e^–^/h^+^ carrier lifetimes in V_o_-rich SrTiO_3–*x*_. The present findings highlight the potential of V_o_-spin engineering toward fine-tuning the electronic properties and photocatalytic activity of perovskite oxides. Technology wise, the present study exemplifies A-FSP as a versatile, industrial scale technology for the synthesis of V_o_-enriched perovskite nanomaterials.

## Graphical abstract

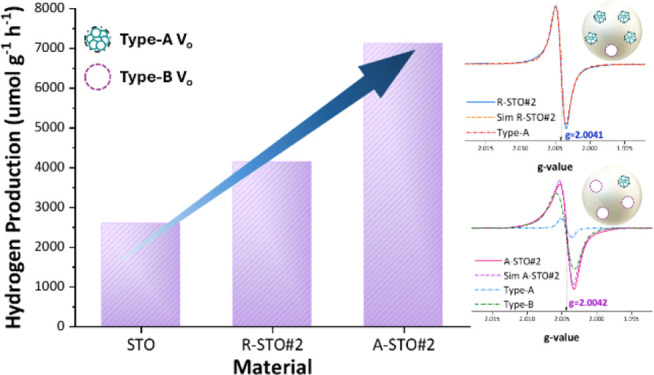

## 1 Introduction

Photocatalytic H_2_ production from H_2_O has emerged as a forward-looking green, environmentally friendly approach to address the current energy crisis. Since 1970, photocatalytic H_2_ production from H_2_O using solar irradiation has demonstrated great potential and has led to extensive research on semiconductor-based materials.^1,2^ Among various photocatalysts, strontium titanate (SrTiO_3_) (STO) is established as a promising Ti perovskite due to its structural stability and favorable energy alignment for photocatalysis, i.e., its conduction band edge is ∼200 mV more negative than that of TiO_2_ ^3^ which enhances the transfer of excited electrons to electron acceptors and possesses a moderate band gap (3.2 eV) photoactivated under UVA-irradiation.^4,5^ So far, numerous efforts have focused on improving the spectral response of SrTiO_3_, e.g., including doping with metals^6,7^ or nonmetals^8,9^ as well as via heterojunction formation with other semiconductors.^10^ However, SrTiO_3_ faces the challenge of a rather high electron–hole (e^–^/h^+^) recombination rate^11^ which significantly hinders its photocatalytic activity, and therefore, the decrease of the (e^–^/h^+^) recombination rate is a key factor to improve the photocatalytic performance.^11^ In this context, oxygen vacancies (V_o_s) may play a key role in the photocatalytic efficiency of SrTiO_3_. In general V_o_s in oxide semiconductors constitute intrinsic lattice defects that may affect decisively the photocatalytic processes.^12–14^

Particularly, in SrTiO_3_, oxygen vacancies have been shown to modulate its electronic structure; thus, if optimized, V_o_s can enhance its photocatalytic activity^15^ and photoelectrochemical (PEC) performance.^16^ Specifically, surface V_o_s can improve the photoinduced (e^–^/h^+^) separation,^12^ adsorption, and activation of H_2_O molecules on the surface, further boosting photocatalytic activity.^17,18^ In contrast, bulk V_o_s appear to function primarily as deep (e^–^/h^+^) traps, leading to an increase in the recombination of photogenerated (e^–^/h^+^) pairs, which in turn diminishes photocatalytic activity.^19,20^ Hence, controlled introduction of V_o_s on a photocatalyst, specifically through simultaneous control of their concentration and spatial distribution, surface versus bulk, is essential for systematically enhancing overall photocatalytic performance.^17,18^

So far, Mao et al.^21^ used high-pressure/high-temperature hydrogenation to reduce TiO_2_, thus creating ‘black TiO_2_’, with a disordered V_o_ layer plus Ti^3+^ centers, leading to improved solar-driven photocatalytic H_2_ activity.^21^ In other pertinent studies, surface V_o_s were shown to promote photocatalytic performance, e.g., in TiO_2_,^22–24^ ZnO,^25,26^ WO_3_,^27^ and CeO_2_.^28^ However, so far, controlled formation of V_o_s in the SrTiO_3_ crystal remains challenging due to its high oxygen binding energy^29^ disfavoring formation of oxygen vacancies.

Usually, SrTiO_3_ can exhibit two kinds of defects i.e. either V_o_s, or reduced metal states (Ti^3+^).^30,31^ It has been reported that creation of Ti^3+^ defects via doping of SrTiO_3_ with low-valence metal cations, e.g., Li^+^, Al^3+^, Mg^2+^, Na^+^, Ga^3+^, and In^3+^ can increase photocatalytic activity^32,33^ via formation of donor energy levels below the minimum of the conduction band.^34^ Domen et al.^35^ have shown that doping of Al^3+^ in SrTiO_3_ exhibits a relatively good H_2_/H_2_O photocatalytic activity, while loading of Rh_2–*y*_Cr_*y*_O_3_ cocatalyst on the Al^3+^-doped SrTiO_3_ can boost the apparent quantum efficiency (AQE) to 30% under 360 nm light.^35^ Following this work, selective photodeposition of Rh/Cr_2_O_3_ and CoOOH cocatalysts enhanced the overall water splitting of Al^3+^-SrTiO_3_, reaching an AQE of ∼96%.^36^ Li et al.^15^ have reported that calcinating SrTiO_3_ of nanofibers under H_2_ (*T* up to 1000 °C) favored the formation of V_o_s, that improved photocatalytic H_2_ production without cocatalyst.^15^ In another work, V_o_-rich disordered SrTiO_3_ surface was produced via reduction of SrTiO_3_, using NaBH_4_ under an Ar atmosphere.^19^ The reduced material exhibited lower energy-gap values with an optimal photocatalytic H_2_ production under ultraviolet–visible (UV–vis) irradiation of 2.2 mmol h^–1^ g^–1^, achieved by a SrTiO_3–*x*_ containing 3.28% (atom) of oxygen vacancies.^19^

Recently, we have demonstrated that anoxic flame spray pyrolysis (A-FSP) technology offers a scalable technology to engineer V_o_s in metal-oxide nanomaterials.^37–39^ Originally, we demonstrated the proof-of-concept in ZrO_2–*x*_ ^38^ and established its application in carbon-coated/Cu_2_O/Cu^0^.^40^ Tehnology wise, to produce SrTiO_3–*x*_ ^39^ perovskite, we introduced CH_4_ as a dispersion gas in the A-FSP process^39^ that enabled controlled synthesis of V_o_-enriched SrTiO_3–*x*_ nanoparticles.^39^ Herein, our focus is to investigate the photophysical and photocatalytic properties of SrTiO_3–*x*_ nanoparticles, focusing on the roles of surface and lattice oxygen vacancies in H_2_/H_2_O photocatalysis.

Our key hypothesis was that surface/lattice V_o_ may affect differently the energy states, band gap, and/or mobility of photoinduced e^–^/h^+^. Herein, to investigate this, we employed electron paramagnetic resonance (EPR) spectroscopy as a state-of-the-art tool to monitor both V_o_s in SrTiO_3–*x*_ and the photogeneration of (e^–^/h^+^) pairs.^41,42^ Recently, we have exemplified the use of EPR to monitor V_o_ dynamics in FSP-made V_o_-rich ZrO_2–*x*_, revealing distinct roles of monomeric V_o_ versus V_o_-clusters in photocatalytic H_2_ production. Here, we apply EPR to A-FSP-made SrTiO_3–*x*_ to study the role of V_o_s and their spin-dynamics in relation to photocatalytic H_2_ production from H_2_O. We show that the V_o_s can intervene in the dynamics of photoinduced (e^–^/h^+^) via magnetic/spin and chemical interactions. Combined with electrochemical impedance spectroscopy (EIS) and Mott–Schottky (M–S) analysis, our study provides new insights into the complex interplay between V_o_s and charge dynamics in SrTiO_3–*x*_.

Specific aims of the present work were [i] to engineer V_o_-enriched SrTiO_3–*x*_ using A-FSP for optimal photocatalytic H_2_ production from H_2_O, [ii] to identify the type of V_o_s in SrTiO_3–*x*_ using EPR spectroscopy in combination with X-ray photoelectron spectroscopy (XPS), [iii] to study the interaction between the V_o_ spins and photoinduced (e^–^/h^+^) pairs using *in situ* EPR spectroscopy and electrochemical analysis, and [iv] to comprehend the complex interplay between V_o_s and the (e^–^/h^+^) pair and H_2_-photogeneration in V_o_-enriched SrTiO_3–_ *x*.

## 2 Experimental and Theoretical Methods

### 2.1 Chemicals and Materials

Ar gas (purity >99%) was purchased from Linde. HPLC grade water was purchased from Merck. Solvents methanol (≥99.9% GC ACS reagent) and 2-propanol (≥99.8% GC ACS reagent) were purchased from Supelco and used without further purification. Dihydrogen hexachloroplatinate (IV) hydrate (H_2_PtCl_6_·*x*H_2_O, 99.9%) was purchased from Alfa Aesar, Nafion solution of 5 wt % perfluorinated Nafion resin solution was purchased from Sigma-Aldrich, 2,2-diphenyl-1-picrylhydrazyl (DPPH) was purchased from Sigma-Aldrich, and sodium sulfate (Na_2_SO_4_, ≥99.9%) was purchased from Merck.

### 2.2 A-FSP Synthesis of SrTiO_3–*x*_ Nanomaterials

The Α-FSP setup and protocols used to produce the STO materials have been described in detail in our previous work.^39^ To provide a comprehensive overview, a schematic diagram of the A-FSP setup is presented in Figure 1. Table S1 in Supporting Information and Table 1 lists all parameters used and the obtained materials. In brief, we have used two Α-FSP setups where the generation of the V_o_s was controlled via injection of a secondary reductive gas, in our case CH_4_, in the combustion process. In the “radial”-A-FSP setup shown in Figure 1a, CH_4_ steam was injected radially along the whole length of the flame with the aid of a perforated enclosing tube. In this setup CH_4_ acts as a diluter of the O_2_ in the whole combustion compartment, promoting the anoxic environment which allows the creation of oxygen vacancies on the SrTiO_3_ nanoparticles. The so-produced SrTiO_3–*x*_ particles are codenamed R-STO# as shown in Table 1.

**Figure 1:**
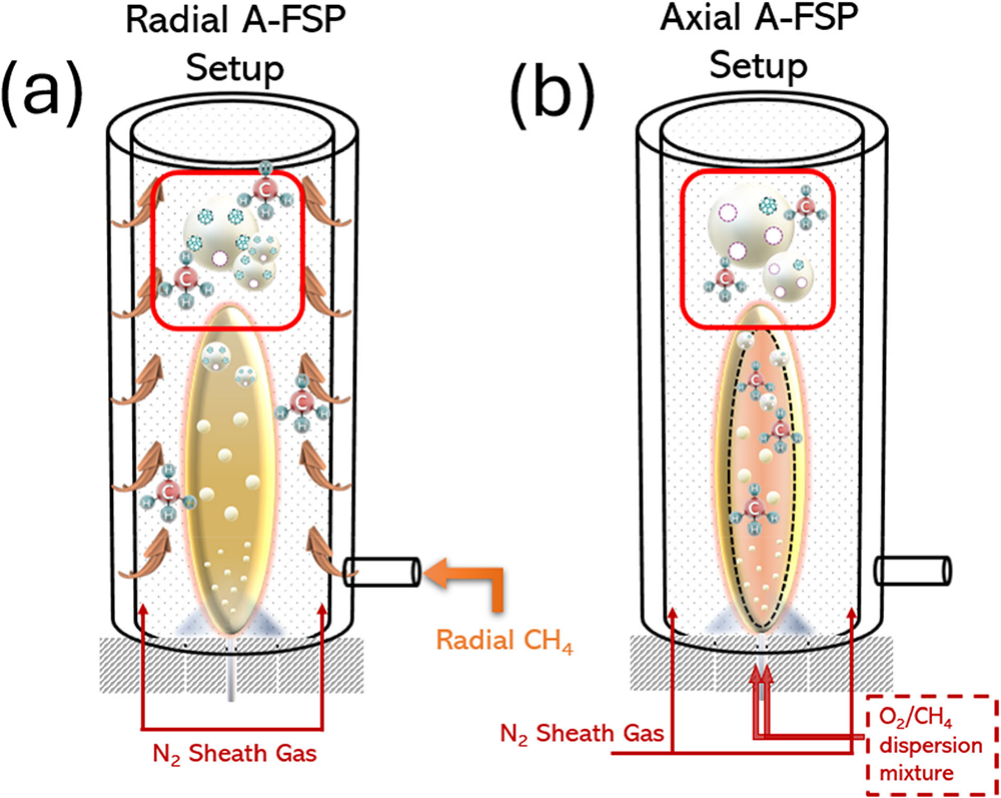
Schematic representation of the A-FSP setup used for the synthesis of (a) radially A-FSP-made and (b) axially A-FSP-made SrTiO_3_ perovskites.

**Table 1:** Axial and Radial CH_4_ Inflows, Calculated XRD Sizes (nm), and FSP F.E.D. (*Ḣ*_C_/*ṁ*_tot,in_) Values

material	CH_4_ inflow (L·min^–1^)	*d*_XRD_ (nm)	F.E.D. *Ḣ*_C_/*ṁ*_tot, in_ (MJ·kg^–1^)^a^
STO	0	39 ± 0.5	11.48
A-STO#1	axial CH_4_: 1	45 ± 0.5	13.95
A-STO#2	axial CH_4_: 2	51 ± 0.5	16.61
R-STO#1	radial CH_4_: 3	43 ± 0.5	11.48
R-STO#2	radial CH_4_: 5	55 ± 0.5	11.48

a F.E.D. (*Ḣ*_C_/*ṁ*_tot,in_) values were calculated usingeq [Disp-formula eq1].

In the second setup, illustrated in Figure 1b, a more drastic intervention toward creating an O_2_-lean FSP combustion environment was implemented by injecting CH_4_ directly into the dispersion gas feed. This approach directly influences the precursor droplets and the nanoparticles at the moment of their formation. By carefully tuning the O_2_-to-CH_4_ ratio in the dispersion gas, we can control the degree of anoxicity within the droplet itself, favoring the formation of bulk V_o_s throughout the particle. Alternatively, adjusting this ratio to create an anoxic atmosphere predominantly surrounding the droplet promotes the formation of surface V_o_s in the resulting nanoparticles. This A-FSP-technology was originally introduced for the controlled production of V_o_-rich ZrO_2–*x*_,^38^ synthesis of non {graphitised C/Cu_2_O/Cu^0^} nanohybrids,^40^ {carbon-SiO_2_ hybrid nanomaterials},^43^ and CeO_2–*x*_.^37^ In this “axial” A-FSP-process, CH_4_ acts as a combustible that consumes O_2_, thus promoting the *in situ* formation of V_o_s during the formation of the primary particles. In the case of “axial”-A-FSP, the injection of CH_4_ is placed in the same stream as the O_2_-dispersion gas, and thus the formed V_o_s are located in the whole volume of the nanoparticles. Herein, the synthesized SrTiO_3–*x*_ particles are referred to as A-STO#X, as listed in Table 1.

A key parameter in the FSP synthesis is the combustion-enthalpy-density, which is defined as the ratio of the combustion enthalpy introduced by the precursor solution, solvent, and methane into the flame (Δ*Ḣ*_C_ in MJ·mol^–1^) to the total inlet mass flow rate, comprising both liquid and gas phases (*ṁ*_tot,in_ in kg·min^–1^), and can be calculated according to Jossen et al. (eq 1):^44^

$$\frac{{\dot{H}}_{C}}{{\dot{m}}_{\mathrm{tot},\mathrm{in}}}=-\frac{\left({\dot{n}}_{\mathrm{precursor}}\Delta {Η}_{c}^{\mathrm{precursor}}+{\dot{n}}_{\mathrm{solvent}}\Delta {Η}_{c}^{\mathrm{solvent}}+{\dot{n}}_{{\mathrm{CH}}_{4}}\Delta {Η}_{c}^{{\mathrm{CH}}_{4}}\right)}{{\dot{m}}_{\mathrm{precursor}-\mathrm{solution}}+{\dot{m}}_{\mathrm{dispersion}\;O_{2}}+{\dot{m}}_{{\mathrm{CH}}_{4}\;\mathrm{flame}}+{\dot{m}}_{O_{2}\;\;\mathrm{flame}}}$$ 1

where *ṅ* and *ṁ* represent the inlet flow rates in mol·min^–1^ and kg·min^–1^, respectively, and Δ*Ḣ*_C_ denotes the combustion enthalpy of each compound. Herein, Δ*Ḣ*_C_ was determined based on the assumption of complete combustion, using the enthalpies of formation (Δ_f_ *H* ^o^) of the reactants and products at 25 °C. It is important to note that eq 1 considers only the enthalpy density supplied directly to the FSP flame, without accounting for ambient air entrainment into the flame.^45^ Therefore, the ratio *Ḣ*_C_/*ṁ*_tot,in_ will hereafter be referred to as the ″FSP feed-enthalpy-density″ or F.E.D. for convenience. The calculated F.E.D. values are listed in Table 1.

### 2.3 Materials Characterization

Powder XRD measurements were collected at room temperature under ambient atmosphere and utilized to investigate the crystal phase and structural characteristics of flame-made STO nanomaterials. Characterization was performed using a Bruker D8 Advance diffractometer equipped with Cu Ka radiation (λ = 1.5405Å). The diffractometer was operated at a current of 40 mA and a generator voltage of 40 kV. To determine the average crystallite size of the nanopowders, Scherrer equation^46^ was employed (eq 2):

$$d_{\mathrm{XRD}}=\frac{K\lambda }{(\mathrm{FWHM})\times \cos\theta }$$ 2

where *d*_XRD_ represents the crystallite size in nanometers (nm), *K* is the shape constant and is equal to 0.9 in our case, λ is the wavelength of the Cu Kα radiation, fwhm is the full-width at half-maximum of the XRD peaks, and θ is the peak diffraction angle.

#### 2.3.1 Electron Paramagnetic Resonance

EPR was employed as state-of-the-art spectroscopy to directly study the surface- and/or bulk-related defects and oxygen vacancies of pristine SrTiO_3_ and V_o_-rich SrTiO_3–*x*_ nanoparticles. A Bruker ER200D spectrometer equipped with an Agilent 5310A frequency counter was used, operating at X-band (∼9.6 GHz) with a modulation amplitude of 10 and 4 G peak-to-peak for the broader and narrower EPR signals, respectively, and a modulation frequency of 100 kHz. The spectrometer was running under a custom-made program based on LabView. To obtain an adequate signal-to-noise ratio, each spectrum is an average of 10 scans. A 20 mg sample was placed into a quartz tube with a 5 mm diameter. Experiments were carried out at 77 K using liquid nitrogen. Prior measurement and each tube was thoroughly degassed using a Kern-vacuum pump for at least 30 min, prior measurement by imersing the EPR tube in a 70 °C water bath, to desorb loosely attached, physiosorbed oxygen without altering the surface structure or inducing additional defects. As verified by EPR spectroscopy, in our systems where the particles are not exposed but to ambient O_2_, this treatment is adequate is generally sufficient to remove any surficial-oxygen EPR signals, e.g., typically detected in the *g* ∼ 2.05 region.

#### 2.3.2 Theoretical Simulation of EPR Spectra

The experimental EPR spectra were simulated using EasySpin MATLAB toolbox^47^ and *S* = 1/2 Spin Hamiltonian (eq 3):

$$H=\beta \vec{B}\cdot g\cdot \vec{S}$$ 3

where β is the Bohr magneton, ***B⃗*** is the applied magnetic field, ***g*** is the *g*-tensor, and ***S⃗*** is the spin angular momentum. Deconvolution and quantitation of the monomeric and cluster V_o_s was done as analyzed previously.^38^ In brief, monomeric V_o_s are identified by their Gaussian lineshape and/or spectrum anisotropy, while the V_o_ clusters are identified by a Lorentz lineshape that originates from the spin–spin interactions between the members of the V_o_ clusters. Further analysis with spin quantitation was performed using DPPH as the spin standard and the double integral of the Gaussian and Lorentzian components.

### 2.4 Photocatalytic Hydrogen Production

Photocatalytic hydrogen (H_2_) evolution experiments were carried out in a double-wall photochemical reactor with a total volume of 340 mL at room temperature (∼25 °C) controlled by an external circulation cooling system. In each experiment, 70 mg of catalyst was suspended in 270 mL of triple-distilled H_2_O and 54 mL of methanol (20% v/v) as the hole scavenger. Atmospheric O_2_ from the solution was excluded by purging the content of the reactor with Ar gas for 45 min. To enhance photocatalytic H_2_ production photodeposition of 1% w/w Pt cocatalyst was implemented using hexachloroplatinate­(IV) hydrate (H_2_PtCl_6_·*x*H_2_O, 99.9%). As the irradiation source, a 250 W mercury lamp was used which was placed inside the quartz immersion well at the geometric center of the photoreactor. The total irradiation power at the average experimental distance of 3 cm was 0.480 mW·cm^–2^ measured with a power meter (Newport, model 1918-C). The produced H_2_ gas was monitored, identified, and quantified every 15 min by a gas chromatography system combined with a thermal conductivity detector (Shimadzu GC-2014, Carboxen 1000 column, argon carrier gas).

### 2.5 Preparation of 1%Pt-SrTiO_3_ Photoelectrodes

#### 2.5.1 Chemicals and Materials

All chemicals were of analytical reagent grade and used without further purification. Fluorine-doped tin oxide (FTO) glass (7 Ω·sq^–1^, transparency 80%, Sigma-Aldrich, USA) was used as the conductive substrate. All aqueous solutions were prepared with HPLC grade water.

#### 2.5.2 Photodeposition of Pt Nanoparticles on SrTiO_3_

The Pt-loaded SrTiO_3_ catalysts were synthesized by a photochemical reduction method. In a typical procedure, 50 mg of SrTiO_3_ was dispersed in an aqueous solution (10 mL) containing 20% (v/v) 2-propanol under vigorous stirring. Then, a nominal amount of H_2_PtCl_6_·*x*H_2_O dissolved in water was added to the suspension described above under stirring. The created mixture was purged with Ar at least 45 min to remove dissolved oxygen. The solution was then irradiated for 1 h under continuous stirring with a Xe 300 W lamp at room temperature. The nominal amount of Pt-loaded catalyst was 1 wt % of the metal.

#### 2.5.3 Preparation of 1%Pt-SrTiO_3_ Photoelectrodes

The SrTiO_3_ films were prepared by the spin-coating method. At first, a slurry was prepared by adding 20 mg of SrTiO_3_ catalysts in a mixture of 1 mL of HPLC grade H_2_O at room temperature, *T* = 23 °C, plus 1 mL of 2-propanol. Then, the catalyst mixture was ultrasonicated for 30 min using a 20 W ultrasonication bath (Elmasonic S10 h, Singen, Germany) to achieve a homogeneous slurry. When homogeneity was achieved, 50 μL of Nafion (sulfonated tetrafluoroethylene-based fluoropolymer-copolymer) solution mixture was added in the mixture and again ultrasonicated for another 40 min, and then the mixture was stirred for 5 h to achieve the final catalyst ink. The Nafion solution consisted of 5 wt % perfluorinated Nafion resin solution in HPLC grade H_2_O/2-propanol (110 uL of Nafion solution, 5.5 mL of HPLC grade H_2_O, and 6.5 mL of 2-propanol). The deposition of each catalyst ink onto the FTO glass (Sigma-Aldrich) was achieved by spin-coating of the prepared homogeneous suspension onto the FTO glass. The film preparation was performed on a SCS6808P spin coater (Specialty Coating Systems, Indianapolis, USA). The thickness of the films was controlled by three factors, first by determining the rotation speed of the FTO glass (rounds per minute), second by the duration of the rotation time, and finally by the drop volume to the FTO glass which was 200 μL. The catalytic ink was spin-coated on the film to achieve a highly homogeneous film. The speed of rotation for all consistent films was selected to be 2500 rpm for 25 s. The film was dried at 50 °C for 1 h and left overnight at room temperature before PEC tests.

#### 2.5.4 PEC Measurements

Analysis of the PEC dynamics for SrTiO_3_, e.g., via EIS and M–S analysis, can provide insightful information on the semiconductor-electrolyte interfacial phenomena and the photoinduced hole–electron dynamics. In this context, herein, we discuss the PEC parameters of our systems on a comparative basis. All PEC measurements were performed on a Corrtest CS2350 electrochemical workstation in a three-electrode cell setup, where spin-coated films acted as the working electrode, a Pt wire as the counter electrode, and Ag/AgCl as the reference electrode. For the PEC measurements, continuous irradiation was used by a 6 W FireJet FJ100 72 × 20 AC, Phoseon Technology Inc. LED source, which emits at 365 nm wavelength, and the intensity of the light source was calibrated with a 1918-C optical power meter (Newport) to simulate AM1.5 illumination (133 mW·cm^–2^). The active area of all photoelectrodes that were exposed to light was 20 cm^2^. In all PEC measurements, the solution was purged with 50 mL·min^–1^ Ar for 45 min to remove dissolved O_2_. PEC measurements were conducted in 0.5 M Na_2_SO_4_ (pH = 7) electrolyte. EIS measurements were carried out in the dark and under illumination at an AC voltage of 10 mV with a frequency region ranging from 0.01 Hz up to 100 kHz at the open-circuit potential (OCP) value. The M–S plots were obtained at a frequency of 1 kHz and an amplitude of 10 mV to determine the flat band potential. All measured potentials were converted to the reversible hydrogen electrode (RHE, pH = 7) according to eq 4:

$$E_{\mathrm{RHE}}=E_{\mathrm{Ag}/\mathrm{AgCl}}+0.059\mathrm{pH}+E_{\mathrm{Ag}/\mathrm{AgCl}}^{0}$$ 4

where *E*_Ag/AgCl_^0^ = 0.1976 V at 25 °C and *E*_Ag/AgCl_ is the measured potential.

## 3 Results and Discussion

### 3.1 Characterization of SrTiO_3–*x*_ Nanomaterials

The XRD patterns of the A-FSP-synthesized SrTiO_3–*x*_, as shown in Figure 2a, exhibit diffraction peaks that correspond well with those of cubic SrTiO_3_ phase (JCPDS card no. 081-9665), consistent with our previous work on the FSP synthesis of these O_2_-deficient STO materials.^39^ The crystallite sizes of SrTiO_3–*x*_ calculated from the XRD data and presented in Table 1 ranged from 39 to 55 nm. A closer examination of the XRD patterns in Figure 2a reveals no observable shift in the 32.4° diffraction peak position. As illustrated in the photographs of Figure 2b, increasing the CH_4_ flow, thereby creating more anoxic conditions during the FSP process leads to noticeable changes in the color of the SrTiO_3_ particles, from white (STO) to gray-yellowish (R-STO#1 and A-STO#1) and finally brown-yellow (R-STO#2 and A-STO#2). This color-darkening trend is due to the formation of oxygen vacancies in the metal-oxide particles made by FSP as observed previously in A-FSP-made ZrO_2–*x*_ ^38^ and SiO_2–*x*_.^43^

**Figure 2:**
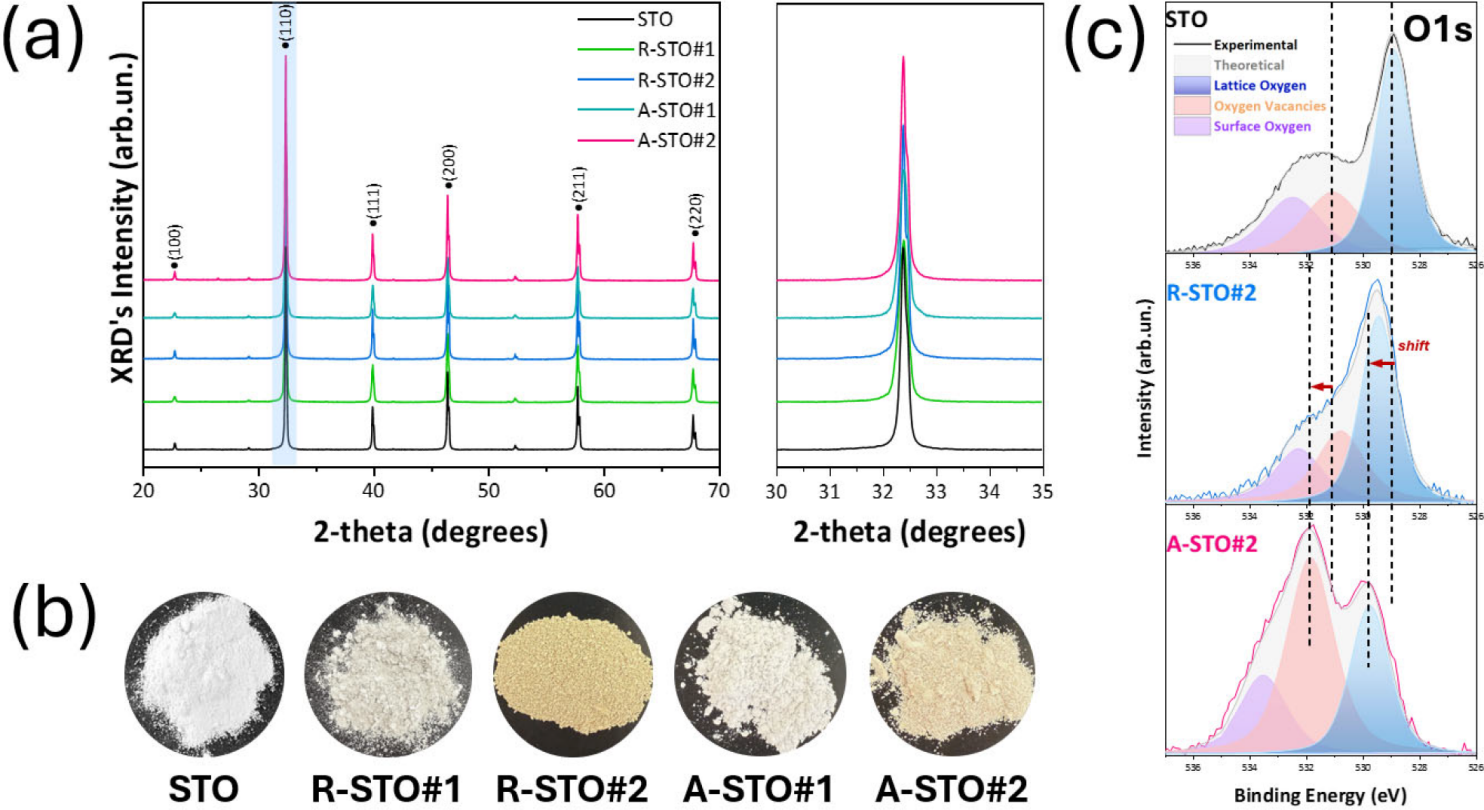
(a) XRD and enlarged XRD patterns in the 2-theta range of 30°–35° of the as-prepared SrTiO_3–*x*_ nanoparticles using the radial (R-STO) and axial (A-STO) A-FSP processes. (b) Photos of the flame-made nanopowders showcasing the color change. (c) O 1s XPS spectra of materials STO, R-STO#2, and A-STO#2.

The O 1s XPS spectra of the STO nanopowders, (Figures 2c and S1) can be deconvoluted into three Gaussian peaks centered at 528.9, 531, and 532.4 eV, respectively. The peak at 532.4 eV is attributed to loosely bound O species on the surface of the STO nanoparticles.^19,48^

The intermediate XPS peak at 531 eV is associated with oxygen vacancies or defective oxygen species.^49,50^ The peak at 528.9 eV corresponds to STO-lattice oxygen within the crystal structure.^49,50^ The XPS data in Figures 2c and S1 indicate distinctly different trends in the lattice-O versus the O-defects, where the introduction of radial CH_4_ in R-STO#1, #2 caused an upshift in the 529 eV peak by 0.5 eV which indicates changes in the STO-lattice oxygens. Additionally, the introduction of axial CH_4_ in the FSP process of A-STO#1, #2 caused significant upshift in the 529 eV peak by >0.8 eV which indicates more severe changes in the STO-lattice oxygen. As expected, axial CH_4_ i.e. where CH_4_ is injected as dispersion gas, affects directly the primary combustion events,^37,38,43^ as reflected in the F.E.D. values shown in Table 1. Apart from the enthalpy, axial CH_4_ provides combustion of CH_4_ inside the flame which depletes O_2_ from the combustion process and, thus, from the *entire structure* of the formed STO particle, and this is evident in the lattice-O 1s XPS for A-STO#1, #2. On the other hand, radial-injection of CH_4_ in the FSP process of R-STO#1, #2 affects the STO particles *after* their formation and during their growth, and thus the effect of O_2_-lean atmosphere is less pronounced in the R-STO, as evidenced in the lattice-O XPS. Here, we should add that XPS primarily detects within a probing depth of approximately 10 nm. Thus, the lattice-O detected in XPS in Figure 2c corresponds to the exterior part of the STO-lattice; thus, they are affected by the milder effect of radial CH_4_ exhibiting a smaller shift in the lattice-O 1s XPS in R-STO compared to A-STO.

The trends observed in the defect-related and surface O 1s XPS spectra (Figures 2c and S1 in Supporting Information) indicate that axial CH_4_ has a significant impact on the formation of oxygen vacancies. This effect is particularly evident in A-STO#2, where increased axial CH_4_ dispersion leads to a pronounced oxygen vacancy signal in the XPS spectrum. This suggests that in A-STO#2, oxygen vacancies are prevalent throughout both the surface and bulk of the SrTiO_3_ particles.

### 3.2 Monitoring V_o_s by EPR Spectroscopy

To further confirm the formation and quantify the concentration of V_o_s in the STO samples, low-temperature (77 K) EPR spectroscopy was employed. A characteristic EPR signal was observed at *g* = 2.0042–2.0044 (Figures 3a–c and S2 of the Supporting Information), typical for spin *S* = 1/2 EPR signals of V_o_s in metal-oxide semiconductors.^38,51,52^

**Figure 3:**
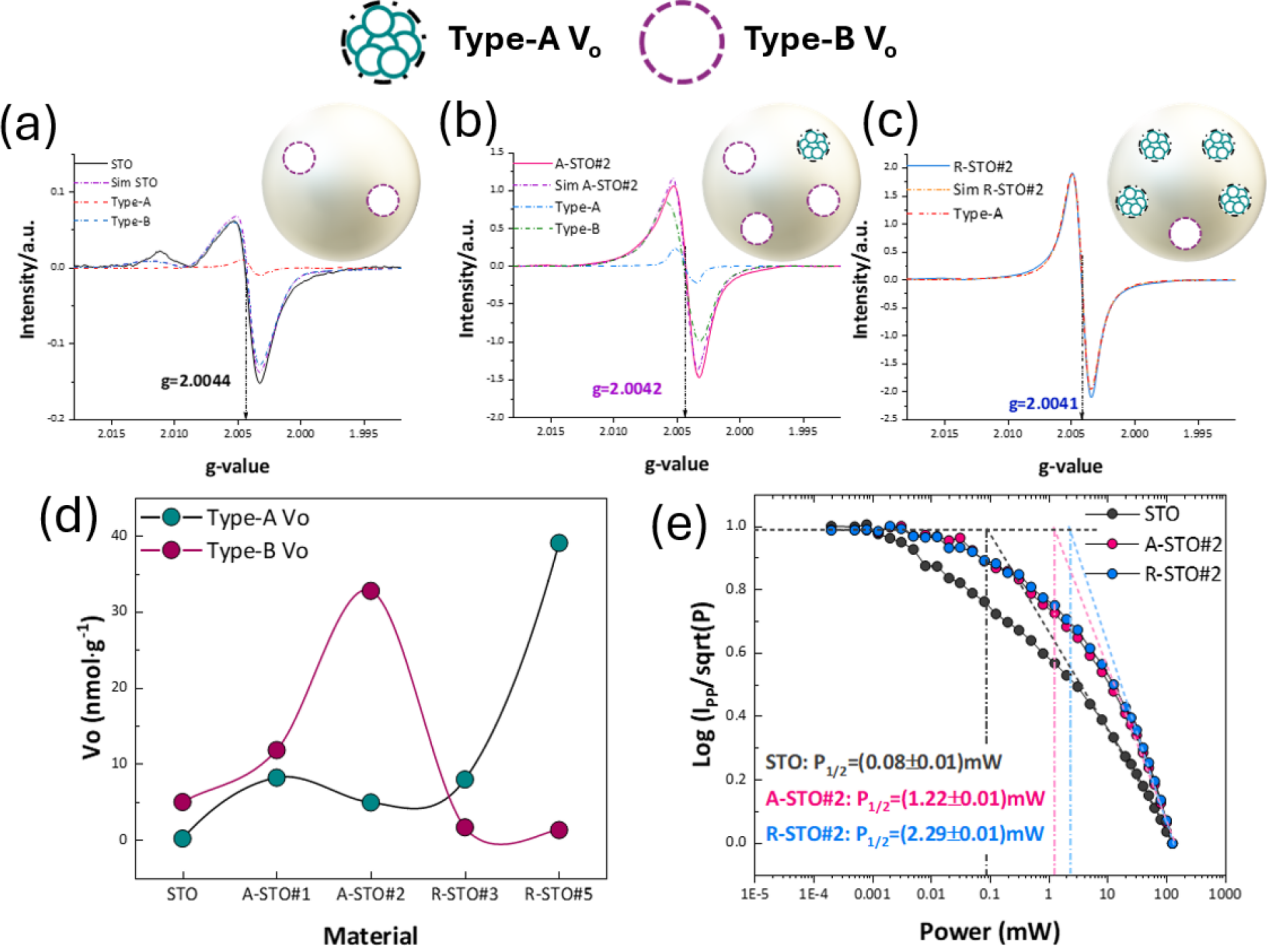
EPR spectra recorded at 77 K for the photocatalysts (a) STO, (b) A-STO#2, and (c) R-STO#2, with the images on the right side of each spectra indicating the vacancy nature. (d) Quantitative analysis and distinction between type-A and type-B V_o_s. (e) Progressive microwave power saturation of EPR spectra of STO, A-STO#2, and R-STO#2, with the images on the right side of each spectra indicating the vacancy nature.

The *g*-values, listed in Table 2, obtained by numeric simulations, show that they do not correspond to carbon radicals, C-centered radicals, which typically exhibit *g*-values in the range of *g* = 2.0018–2.0028.^53^ The absence of carbon in our STO -materials is further confirmed by Raman data. Specifically, the absence of Raman bands at 1350 and 1590 cm^–1^ correspond to the D- and G-bands of carbon, respectively (see Figure S3 in Supporting Information).

**Table 2:** Spin Hamiltonian Parameters of Materials STO, A-STO#1, #2, and R-STO#1, #2

material	[*g*_*x*_, *g*_*y*_, *g*_*z*_] ± 0.0002 (type-A)	[*g*_*x*_, *g*_*y*_, *g*_*z*_] ± 0.0002 (type-B)	type-A (% ± 1)	type-B (% ± 1)
STO	[2.0045 2.0035 2.0039]	[2.012 2.0076 2.0078][2.0045 2.0035 2.00398]	5	95
A-STO#1	[2.0051 2.0033 2.0042]	[2.0037 2.0036 2.0071]	48	52
A-STO#2	[2.0055 2.0036 2.0046]	[2.0042 2.0042 2.0062]	13	87
R-STO#1	[2.0032 2.0041 2.0050]	[2.0037 2.0052 2.0064]	82	18
R-STO#2	[2.0039 2.0039 2.0049]	-	99	1

Overall, based on the present EPR, XPS, and Raman data, we conclude that the EPR signals detected in FSP-made STO materials in Figures 3 and S2, S3 in Supporting Information correspond to V_o_ centers. We notice that the pristine FSP-made STO also contains some V_o_s as evidenced by EPR and XPS. These are ‘intrinsic” V_o_s inherently formed at low contractions during the FSP combustion/particle formation process.

As we have shown previously,^38^ theoretical analysis of the lineshapes of the Vo-EPR spectra can provide valuable information on the monomeric and cluster character of the V_o_s in the nanoparticles. Accordingly, a numerical combination of Lorentzian and Gaussian lineshapes was used to account for both homogeneous and inhomogeneous broadening. As shown in Table 2, the Lorentzian component is codenamed as type-A and the Gaussian component as type-B. The type-A Lorentz lineshape is defined by strong, sharper, symmetric EPR signals with long-decaying tails, indicative of homogeneous broadening^38^ that originates from spin–spin interactions. In the case of V_o_ in nano-oxides, the type-A Lorentz lineshape originates from closely spaced large population of clustered V_o_. On the other hand, type-B Gauss lineshape is defined by broader and/or anisotropic EPR signals indicative of inhomogeneous broadening that originates from isolated spins. In the case of V_o_ in nano-oxides, the type-B Gauss lineshape originates from monomer centers with no nearby paramagnetic centers.

Within this context, a theoretical estimation of the Lorentzian-to-Gaussian ratio L/G was performed for the EPR signals of our STO materials. As listed in Table 2, pristine STO exhibited L/G = 0.05, indicating that the V_o_s in STO are almost 100% type-B V_o_. On the other hand, the R-STO#2 sample exhibited L/G = 0.99 indicating that the V_o_s in R-STO#2 are almost 100% type-A V_o_s. The A-STO#2 sample had L/G = 0.13, suggesting a mixture of monomeric/cluster V_o_s. Additionally, regarding pristine STO, the component at *g* ≈ 2.01 is attributed to type-B oxygen vacancies, monomeric, well-dispersed defects characterized by a Gaussian lineshape arising from inhomogeneous broadening. The observed secondary peak at slightly higher *g* values (*g* > 2.01) is indicative of anisotropic V_o_ centers, likely situated in a locally distorted lattice environment, which further supports the presence of structural asymmetry around these vacancy sites.

Based on the work of Esch et al. the nature of oxygen vacancies in nano-oxides is closely related to the calcination temperature.^54^ Direct diffusion of oxygen vacancies on the CeO_2_ surface required temperatures higher than 400 °C, thus giving low possibility of single oxygen vacancies accumulation and cluster formation. As a result, the oxygen vacancies in this sample were primarily observed as single vacancies. However, calcination of the samples at 450, 550, and 650 °C facilitated the diffusion of oxygen vacancies, leading to the formation of predominant oxygen vacancy clusters in these CeO_2_ samples.^54^ In the present case, of A-FSP synthesis of STO, and particularly in the axial A-FSP process, the incorporation of CH_4_ into the dispersion feed resulted in a significant increase in the combustion-enthalpy-density. Using eq 1, we calculated the F.E.D. values for the A-FSP process, which are summarized in Table 1. For pristine STO, the F.E.D. value was 11.48 MJ·kg^–1^, increasing to 13.95 and 16.61 MJ·kg^–1^ for A-STO#1 and A-STO#2, respectively. According to the principles of FSP process,^44,55^ this trend indicates that upon increase in the enthalpy feed density, an elevated FSP-flame temperature profile is attained, which prolongs the high-temperature-particle-residence-time. In contrast, for the materials R-STO#1 and R-STO#2, the corresponding FSP F.E.D. values remain equivalent to that of pristine STO (F.E.D. = 11.48 MJ·kg^–1^) i.e. as the radially introduced CH_4_ does not directly affect the flame but rather modifies, in a secondary manner, the combustion environment through which the STO-formed particles evolve until they are collected on the glass fiber filter.

Quantitative analysis of the two types of V_o_s based on the EPR data is provided in Figure 3d. We observe progressive increase in the type-B V_o_ population for materials A-STO#1 and #2, while materials R-STO#1 and #2 exhibit higher amounts of type-A V_o_s. The lattice dynamics of STO, A-STO#2, and R-STO#2 were studied by the microwave EPR saturation technique^56,57^ (Figure 3e). For convenience, full EPR data for each material are summarized in Figure S4 of the Supporting Information. The progressive microwave saturation EPR data offer insights into the spin-relaxation mechanisms that govern the spin–lattice dynamics of the V_o_ centers as monitored via their EPR signal.^58,59^ When the microwave power reaches a saturating level, the V_o_ spin system can no longer dissipate it through coupling with the lattice. As a result, the EPR signal intensity decreases rapidly, as shown in Figure 3e. The key information is the so-called microwave saturation power (*P*_1/2_) values that is calculated as the inflection point in Figure 3e. *P*_1/2_ value represents the microwave power a spin system can dissipate at equilibrium via lattice interactions. Stronger coupling between the spins and lattice vibrations corresponds to a higher *P*_1/2_ value and *vice versa*. The *P*_1/2_ data in Figure 3e reveal that the V_o_s in the A-STO#2 and R-STO#2 materials are in close magnetic interaction with the particle lattice as evidenced by the increased *P*_1/2_ values of 1.22 mW for A-STO#2 and 2.29 mW for R-STO#2 compared to the much lower value of 0.08 mW for pristine STO. Taking into account the Lorentz/Gauss data, we conclude that the type-A Lorentz V_o_ *clusters* are the origin of the high *P*_1/2_ dynamics. This analysis reveals a novel aspect of V_o_s in the SrTiO_3–*x*_ nanolattice, which in structural terms indicates that the type-A vacancies are closely associated groups located in the same domain of the particle, and they constitute clusters of V_o_s. This is in agreement with their Lorentz EPR lineshape.

Overall, the present EPR data clearly show that the SrTiO_3–*x*_ particles contain two types of V_o_s: type-A V_o_-clusters which form as localized agglomerates in the nanomatrix and have strong magnetic interactions, and type-B monomeric V_o_ that are isolated defects well dispersed in the STO matrix.

#### 3.2.1 Photocatalytic Dynamics: H_2_ Production Efficiency and Hole–Electron Pair Dynamics

Figure 4a illustrates the photocatalytic H_2_ production from H_2_O by the STO materials, all of which exhibited a linear increase in H_2_ production over the course of 3 h. The corresponding H_2_ production rates (expressed in μmol·g^–1^·h^–1^ are displayed as bars in Figure 4b) indicate that the A-STO materials demonstrate a significantly enhanced photocatalytic performance. In particular, A-STO#2 achieved a production rate of 7000 μmol·g^–1^·h^–1^, representing an approximate 200% increase compared to that of pristine SrTiO_3_. Superposition of the V_o_ data (Figure 4a), from EPR in Figure 3d, reveals a clear trend where the H_2_ photoactivity is boosted by the type-B monomeric V_o_s. Materials A-STO#1 and A-STO#2 with a predominance of type-B V_o_s are superior photocatalysts, while materials R-STO#1 and R-STO#2 which display a predominance of type-A over type-B vacancies show only a marginal improvement in H_2_ production (Figure 4b). These findings concur with previous results in anoxic ZrO_2–*x*_ where monomeric V_o_s were shown to be preferable boosters of the photocatalytic H_2_ production.^38^

**Figure 4:**
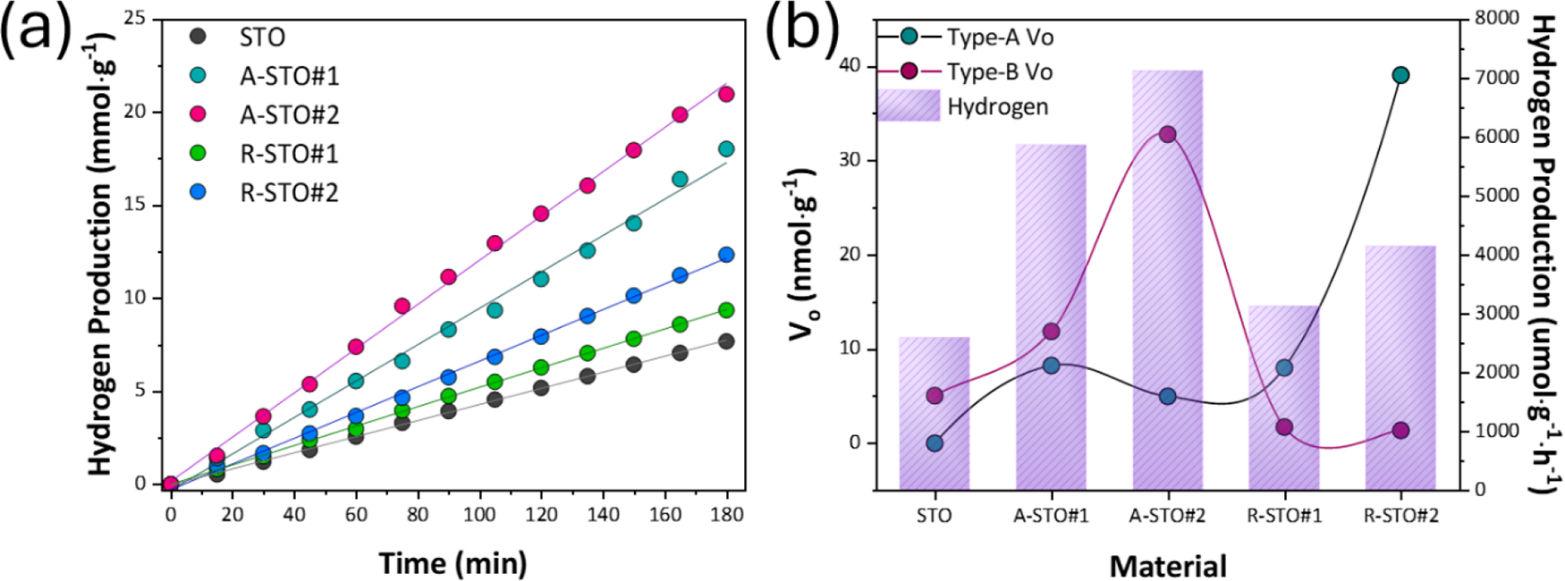
(a) Photocatalytic kinetics of H_2_ production under UV irradiation. (b) Correlation plot of the detectable V_o_-EPR signals (type-A and type-B) quantified in nmol·g^–1^ of material using DPPH versus H_2_ production rate in μmol·g^–1^·h^–1^.

Figure 4a,b present the EPR signals of the photoinduced electrons and holes formed *in situ* in the STO crystals. In Figure 5a, the three observed EPR signals with *g*-values of 1.995, 1.978, and 1.922 are attributed to photoinduced electrons^–60^.^61^ In TiO_2_, such e^–^ are typically localized on the Ti^3+^ centers. In SrTiO_3_, these signals are associated with responses from different types of Ti centers, octahedral sites within a distorted oxygen environment,^60^ or octahedral sites with two oxygen vacancies.^61^ The photoinduced h^+^ are typically resolved *g*-values >2, as shown in Figure 5b. Based on this assignment, in Figure 5c, we present a plot of the populations of the photoinduced e^–^ and h^+^. Strikingly, we observe that material A-STO#2 which produced the highest H_2_ has one of the lowest detectable photoinduced e^–^ signals. Similarly, there appears to be a trend toward decrease of the detectable photoinduced e^–^ signals for all SrTiO_3–*x*_ materials compared to STO.

**Figure 5:**
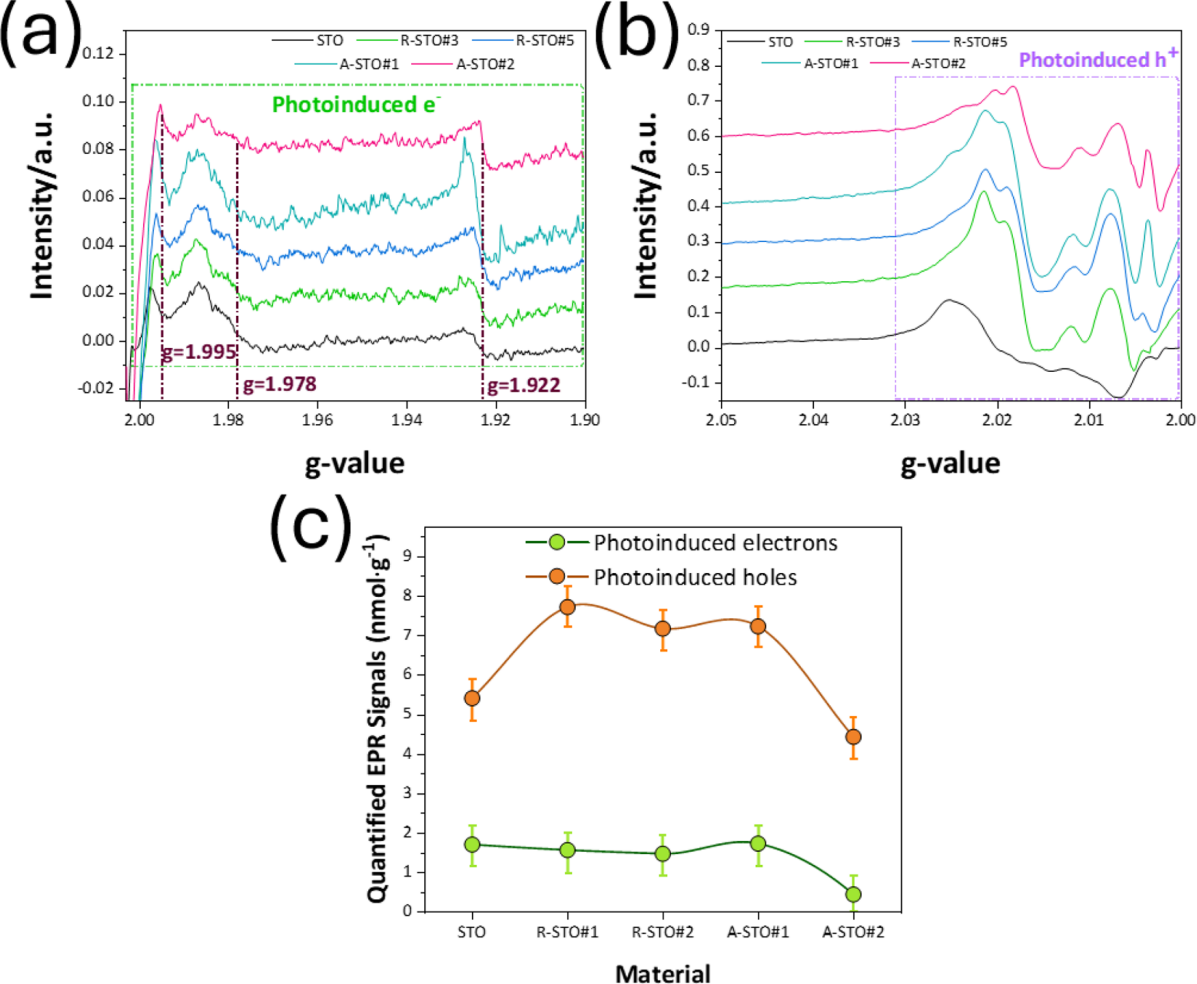
EPR spectra recorded at 77 K of photoinduced (a) e^–^ and (b) h^+^ of the as-prepared STO nanopowders. (c) Quantified photoinduced (light minus dark) signals (e^–^/h^+^) in nmol·g^–1^ of material using DPPH. The introduction of CH_4_ either axially or radially has a profound effect on the photoinduced e^–^ population.

This apparent suppression of the EPR signal intensity, primarily for the photoinduced electrons, is in disparity with the observed H_2_ production trends. Material A-STO#2 with the highest H_2_ production shows the lowest EPR-detectable {e^–^}. EPR signals can lose intensity when broadened via magnetic interactions with nearby steady-state paramagnetic entities in the same nanomatrix. We consider that the V_o_s in SrTiO_3–*x*_ are the steady paramagnetic entities that broaden the photoinduced {e^–^} signals. Interestingly, the EPR h^+^ appear to be less affected by the paramagnetic centers in the STO materials, which might be related to the more localized character of the holes in Ti-based photocatalysts.^62^

Overall, our EPR spectroscopy data provide critical insight into the multifaceted role of V_o_s in modulating photocatalytic H_2_ production. Low-temperature (77 K) EPR analysis identified two distinct types of V_o_ centers: Type-A (clustered vacancies with Lorentzian line shape) and type-B (monomeric vacancies with Gaussian line shape). Type-A V_o_s correspond to closely spaced vacancy clusters within the SrTiO_3_ lattice, leading to strong spin–spin interactions and significant spin–lattice coupling. These localized defect agglomerations facilitate rapid recombination of photogenerated electron–hole pairs, thereby suppressing photocatalytic efficiency. In contrast, type-B V_o_s are well-dispersed, isolated defects that exhibit minimal magnetic coupling. Functioning as shallow electron traps, they enhance charge separation by stabilizing photogenerated electrons and mitigating recombination. This mechanistic distinction is further supported by power saturation EPR analysis, which reveals that type-A V_o_s require higher microwave saturation power due to their stronger lattice coupling, while type-B V_o_s remain weakly coupled and thus favor efficient charge-carrier mobility. Notably, materials enriched in type-B V_o_s, such as A-STO#2, exhibited the highest photocatalytic H_2_ evolution rates, reaching up to 7000 μmol·g^–1^·h^–1^, highlighting a direct correlation between the monomeric V_o_ concentration and photocatalytic performance. Conversely, samples dominated by type-A V_o_s, such as R-STO#2, showed reduced H_2_ production, consistent with the recombination prone nature of vacancy clusters. Collectively, these findings underscore that the nature, distribution, and magnetic behavior of oxygen vacancies are decisive in governing the charge dynamics and photocatalytic efficiency of SrTiO_3–*x*_ systems.

Taking all this information into account, it is revealed that in anoxic V_o_-rich STO, vacancies play multiple/critical roles. First, they determine the optimal lattice configuration that maximizes photocatalytic H_2_ production, as evidenced by the V_o_-rich A-STO#2 sample, which exhibits the highest photocatalytic H_2_ production. Second, vacancies influence the spin dynamics of photogenerated e^–^ and h^+^; at high oxygen vacancy concentrations, e^–^ become undetectable by EPR, as shown in the schematic representation of Figures 5; however, they are efficient in H_2_ formation. Finally, when the STO-lattice becomes dominated by vacancies, the e^–^-mobility is severely impaired, leading to a significant reduction in H_2_ production. These perplexing aspects are schematically summarized in Figure 6.

**Figure 6:**
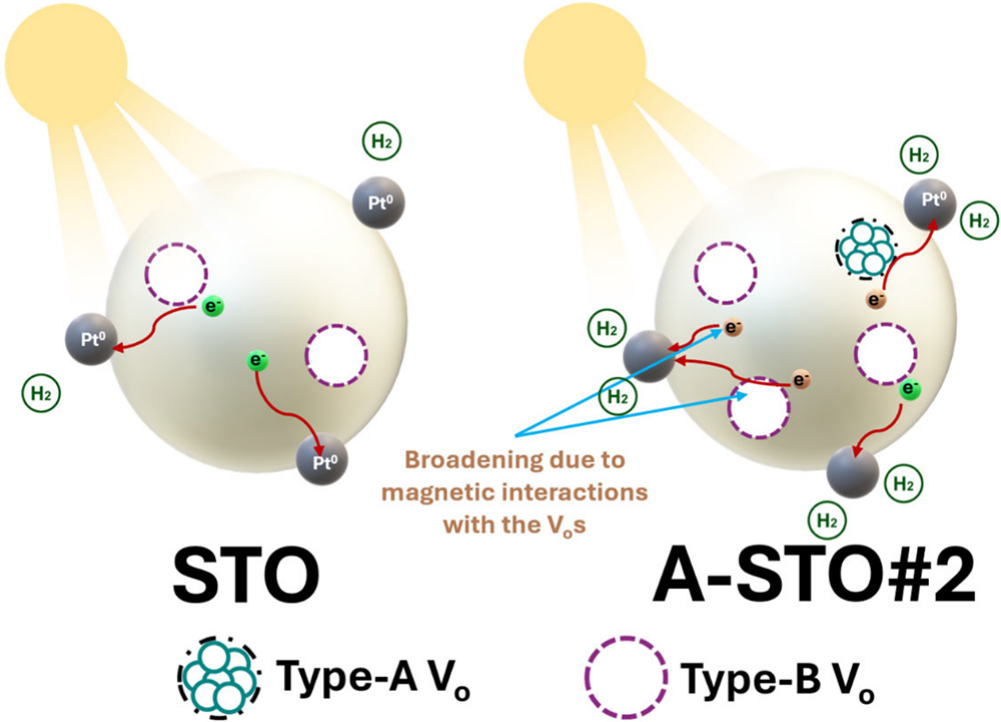
Schematic representation of the STO and A-STO#2 materials under illumination. The enhanced anoxicity induced by the axial A-FSP process results in increased H_2_ production, accompanied by a higher concentration of monomeric oxygen vacancies, as detected by EPR spectroscopy. Interestingly, this high H_2_ production is anticorrelated with the EPR-detectable photoinduced e^–^/h^+^ signals.

To further consolidate these observations and to peer into the perplexing role of V_o_ in the {e^–^/h^+^} dynamics and photocatalytic H_2_ production performance, we performed a PEC EIS and M–S study.

### 3.3 Photo Electro Chemical Dynamics of SrTiO_3_ Nanomaterials

#### 3.3.1 EIS Analysis

EIS measurements were conducted to study the role of V_o_s of SrTiO_3‑x_ in the charge-transfer rate between the semiconductor and the solution interface. Figure 7 presents the EIS data in the dark or under irradiation, denoted as “light off” and “light on,” as well as their respective simulated Nyquist plots with dashed and dotted lines according to the values of Table S2, respectively, versus OCP.

**Figure 7:**
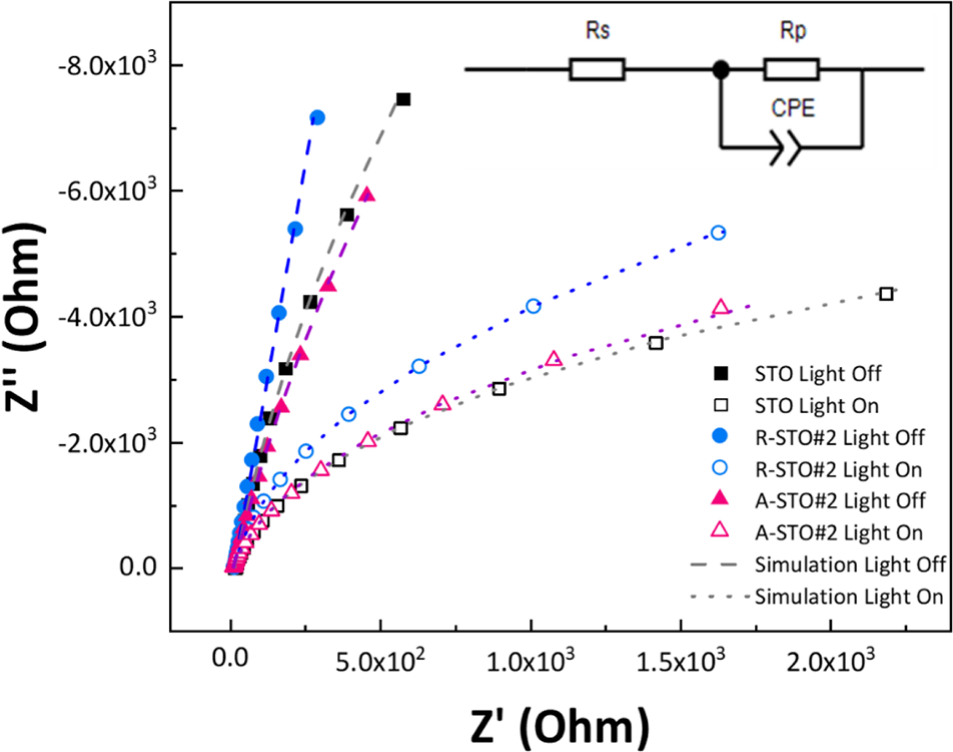
EIS plots of STO materials performed in 0.5 M Na_2_SO_4_ solution in dark (light off) condition or under ultraviolet irradiation (UV-light) (light on) condition versus the case of the OCP. The equivalent circuit is presented as the inset figure, which is used to calculate the double-layer capacitance and the resistance of semiconductor/solution, which are responsible for the charge-transfer phenomena. Dashed and dotted lines represent the simulated Nyquist plots corresponding to the fitted EIS data for the respective samples.

The equivalent circuit shown in the inset in Figure 7 was employed for the analysis of EIS data. This circuit comprises *R*_s_, representing the solution resistance, connected in series with *R*_p_, which denotes the charge-transfer resistance, in parallel with a constant phase element (CPE).^63^ The CPE is characterized by two parameters: CPE-T (magnitude) and CPE-P (the phase exponent). When CPE-P equals 1, the CPE functions as an ideal capacitor, with CPE-T corresponding to its capacitance.^63^ For values of CPE-P ranging from 0.9 to 1, the system exhibits behavior indicative of double-layer capacitance, functioning as a CPE. This behavior is observed in the materials under study, as evidenced by the data presented in Table S2 of the Supporting Information. The solution resistance *R*_s_, as listed in Table S2, remains nearly constant across all of the samples. According to the EIS results, the photocatalyst exhibiting the smallest semicircular arc under illumination is in the order: A-STO#2 > R-STO#2 > STO (Figure 7). The observed differences in arc diameter are primarily attributed to variations in *R*_p_ and the associated CPE parameters, which are influenced by the V_o_s. These vacancies enhance interfacial charge transfer, contributing to more efficient separation and mobility of photogenerated charge carriers. This trend correlates well with EPR that shows that A-STO#2 possesses the highest concentration of type-B vacancies, followed by R-STO#2 and then pristine STO. Notably, the values of *R*_p_ follow the same sequence, reinforcing the connection between the surface defect density and the charge-transfer behavior. The enhanced {e^–^/h^+^} separation of A-STO#2 significantly improves its photocatalytic performance in hydrogen evolution. The *R*_p_ value indicates superior efficiency in facilitating charge-carrier transport and suppressing recombination, thereby enabling the highest hydrogen evolution activity among the samples studied.^19^

#### 3.3.2 Mott–Schottky Analysis

For the M–S plots, the space-charge capacitance (*C*_SC_) of the electrode/electrolyte interface was measured in 0.5 M Na_2_SO_4_ (pH = 7) electrolyte. The plots were obtained at a frequency of 1 kHz and an amplitude of 10 mV peak–peak to determine the flat band potential. The solid/electrolyte interface can be conceptualized using a three-layer model.^64,65^ The total capacitance at this interface is the series combination of the capacitances of these layers: 1/*C* = 1/*C*_SC_ + 1/*C*_H_ + 1/*C*_G_,^64^ where *C*_SC_ is the space-charge region (SC) in the semiconductor, *C*_H_ is the Helmholtz layer, and *C*_G_ is the diffuse ionic layer in the electrolyte. Due to their relatively small thickness, the contributions from the diffuse and Helmholtz layers are often negligible; thus, we approximate 1/*C* ≈ 1/*C*_SC_. From EIS, the total capacitance of the solid/electrolyte interface can be estimated, and using this, the so-called M–S plot^66^ can be constructed, i.e., *C* ^2–^ as a function of the applied voltage potential *V*_app_. Additionally, the oxygen deficiency of those photocatalysts is directly related to the electron density; therefore, another key tool to evaluate these materials is by investigating their flat band potentials (*V*_fb_) through M-S analysis. The *C*_SC_ depends on the flat band potential *V*_fb_ according to eq 5,

$$\frac{1}{C^{2}}=\frac{1}{C_{\mathrm{SC}}^{2}}=\frac{2}{e\varepsilon \varepsilon_{0}N_{D}A^{2}}\left(V_{\mathrm{app}}-V_{\mathrm{fb}}-\frac{k_{B}T}{e}\right)$$ 5

where *e* is the electron charge, ε is the dielectric constant of the semiconductor, ε_0_ is the vacuum permittivity, *N*_D_ is the donor concentration in the semiconductor, *A* is the area, *V*_app_ is the applied potential, *V*_fb_ is the flat band potential, *k*_B_ is the Boltzmann’s constant, and *T* is the absolute temperature. The donor density (*N*_D_) of the materials was estimated from the slope of linear portion of the M–S plots according to the following eq 6:

$$N_{D}=\frac{2(V_{\mathrm{app}}-V_{\mathrm{FB}})\cdot C_{\mathrm{SC}}^{2}}{\varepsilon \varepsilon_{0}e}$$ 6

where *N*_D_ is the donor density of the electrode material, and the other variables are the same with equation *Y*, where ε_0_is 8.8542 × 10^–10^ F cm^–1^, *e* is 1.602 × 10^–19^ C, and ε is 340 for SrTiO_3_ according to literature reports.^67^. The term (*V*_app_ – *V*_FB_)·*C*_SC_^2^ is the reciprocal of the slope (α) of the M–S plots, and therefore it can be simplified as shown in the eq 7:

$$N_{D}=\frac{4.147\times 10^{25}}{a}\left(\frac{\mathrm{cm}}{F\cdot C}\right)$$ 7

Figure 8 displays the M–S plots obtained for thin films fabricated using the SrTiO_3_ nanoparticles synthesized in our previous study,^39^ providing insights into their semiconductor properties.

**Figure 8:**
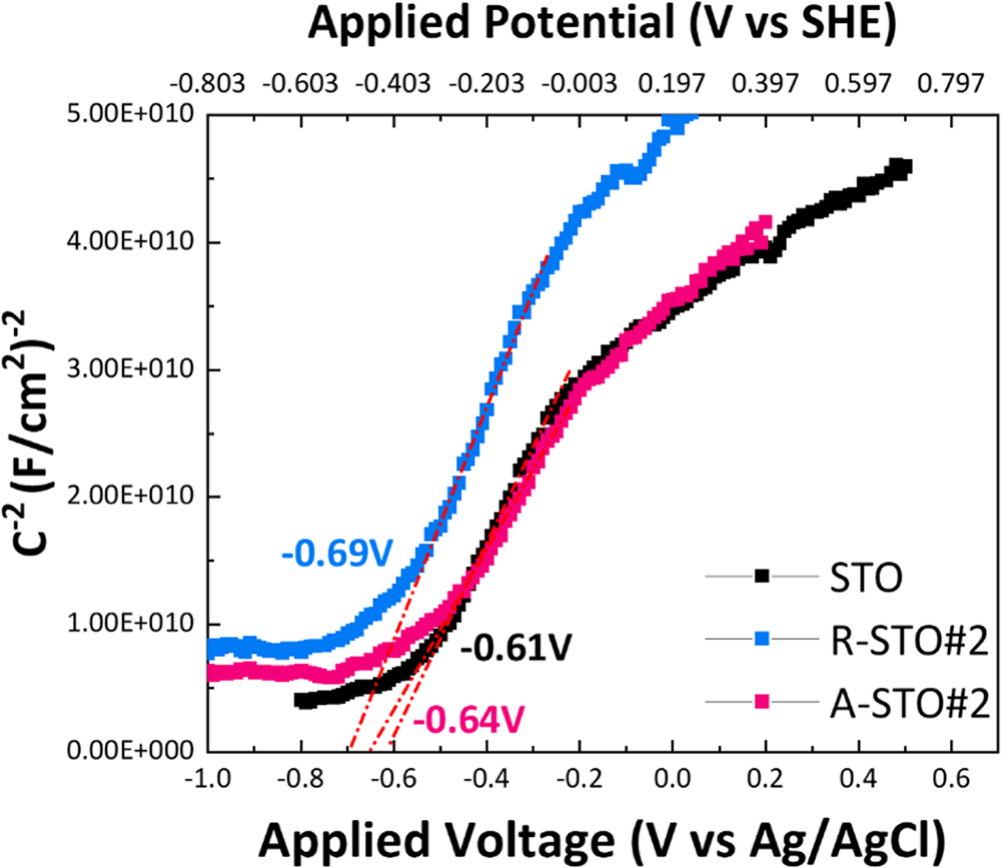
M–S plots for the nanocatalysts in 0.5 M Na_2_SO_4_ performed under UV irradiation. The plots were obtained at a frequency of 1 kHz and an amplitude of 10 mV peak-to-peak to determine the flat-band potential.

The M–S plots in Figure 8 have positive slopes, which indicate that all samples are n-type. *V*_fb_ can be read by extrapolating the M–S plot to the *x*-axis where 1/*C* ^2^ is equal to zero. When applying a more positive potential (*V*_app_ > *V*_fb_), the electrode (*V*_app_ > *V*_fb_)­described as a Schottky junction forms space-charge (depletion) layer with a width (*W*), as described in eq 8):

$$W=\sqrt{\frac{2\varepsilon \varepsilon_{0}(V_{\mathrm{app}}-V_{\mathrm{fb}})}{eN_{D}}}$$ 8

Thus from the data in Figure 8, using eq 6–8 the so-estimated values of flat band potential values, the slope, the donor density, and the depletion layer for these materials are presented in Table 3. It is known that for n-type semiconductors, the conduction band (*E*_CB_) is above the flat band potential of about 0.1–0.2 V.^40^ Accordingly, the flat band potential (*V*_fb_ vs Ag/AgCl) is estimated to be −0.61, −0.64, and −0.69 V for STO, A-STO#2, and R-STO#2, respectively. Ensuingly, the *E*_CB_ was estimated as −0.81, −0.84, and −0.89 V vs Ag/AgCl for STO, A-STO#2, and R-STO#2, respectively. This analysis shows that R-STO#2 has the most negative *E*_CB_ that is more reducing in H_2_ production.

**Table 3:** Calculated Values of Flat Band Potential (*V*_fb_), Reciprocal of the Slope (α), Donor Density (*N*_D_), and Depletion Layer (W) via M–S Analysis

photoelectrodes	*V*_fb_ (V vs Ag/AgCl)	α	*N*_D_ (× 10^15^ a/cm^3^)	*W* (nm)@0 V vs Ag/AgCl
STO	–0.61	1.2219 × 10^11^	5.43	3.88
A-STO#2	–0.64	9.944 × 10^10^	6.67	4.80
R-STO#2	–0.69	6.414 × 10^10^	10.3	5.84

According to Table 3, the charge-donor density for each material is in the range of 5.4 × 10^15^ up to 10.3 × 10^15^ A/cm^3^. The depletion layer width *W* at 0 V vs Ag/AgCl, fort R-STO#2 has the highest value of 5.84 nm, due to the high amount of donors, almost 1 order of magnitude versus the other materials, A-STO#2 has *W* equal to 4.80 nm, which is a high value due to the high value of flat band potential which boosts the width of depletion, while STO has the lowest value due to the fact that it has both low amount of donors and the most positive flat band potential.

#### 3.3.3 {e^–^}–{h^+^} Recombination Dynamics Analysis

According to the studies conducted by Kim et al. and Li et al.^19,68^ Open circuit potential (OCP) decay profiles can be recorded by terminating the light irradiation. This approach enables the investigation of charge-carrier recombination dynamics, and—in our present case—how these are influenced by the presence of oxygen vacancies of various types. The recombination rate can be fitted using first-order kinetics described by eq 9:

$$\frac{V-V_{\mathrm{on}}}{V_{\mathrm{off}}-V_{\mathrm{on}}}=1-e^{\left(-k_{r}t\right)}$$ 9

where *V* is the measured potential at any time, *V*_on_ and *V*_off_ are the OCP values under light-irradiation and dark condition, respectively, *k*_r_ is the pseudo-first-order recombination rate constant, and *t* is the time. Figure 9 shows the OCP decay profiles of STO, R-STO#2, and A-STO#2.

**Figure 9:**
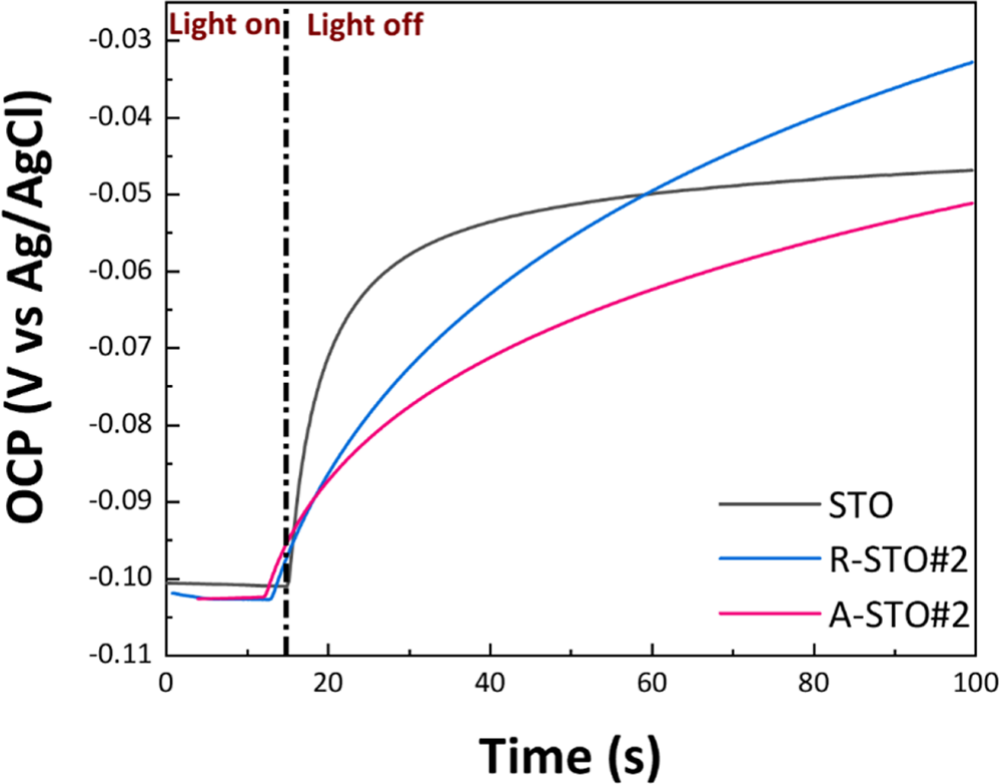
OCP decay curves were performed in 0.5 M Na_2_SO_4_ solution. First, all samples were under irradiation until the OCP potential was stabilized, and then we switched them to dark conditions and measured the OCP decaying.

As shown in Figure 9, pristine STO exhibits the fastest charge-carrier recombination, while the other two materials demonstrate slower recombination dynamics, with A-STO#2 exhibiting the longest recombination time. Accordingly, the present OCP decay analysis shows that in the case of STO, electrons are primarily excited within the bulk under irradiation and subsequently recombine with holes, resulting in only a limited portion of photogenerated electrons contributing to photocatalytic hydrogen production. In contrast, for A-STO#2, photogenerated electrons are effectively stabilized at V_o_ sites, which are considered to act as active sites for the hydrogen evolution reaction (HER). This trapping minimizes the {e^–^/h^+^} recombination rate, thus increasing the availability of electrons for photocatalysis. Consequently, the number of electrons that recombine in A-STO#2 is significantly lower than the initial number of photogenerated charge carriers as a portion of them is utilized in HER.

Overall, the present EPR and electrochemical data reveal that type-B oxygen vacancies, corresponding to monomeric isolated defects, enhance the performance of SrTiO_3–*x*_ through three distinct mechanisms: first, type-B V_o_s induce subtle local distortions in the SrTiO_3_ lattice, which result in the formation of additional electronic density of states (DOS) within the band structure. These defect-induced states can act as shallow traps that facilitate the capture and transfer of photogenerated electrons, thereby promoting more effective charge separation and suppressing recombination. Second, these vacancies modulate the overall electron mobility within the SrTiO_3_ matrix. By introducing localized states close to the conduction band, type-B V_o_s provide pathways that can enhance carrier dynamics. This improved mobility enables more efficient migration of photogenerated electrons to the catalyst surface, where they can participate in reduction reactions, such as hydrogen evolution. Finally, type-B V_o_s are typically formed at relatively low concentrations which ensures that they do not compromise the structural integrity or dynamic stability of the SrTiO_3_ lattice. The presence of dispersed monomeric vacancies preserves the crystallinity and minimizes the adverse effects on the semiconductor’s bulk properties.

## 4 Conclusions

In the present work, we systematically investigated the role of lattice oxygen vacancies in modulating the H_2_-photocatalytic efficiency *in tandem* with the {e^–^/h^+^} spin-pair dynamics in V_o_-rich SrTiO_3_ nanoparticles synthesized by FSP. By controlling the feed-enthalpy-density of the A-FSP process, we can selectively tune the nature and concentration of oxygen vacancies as confirmed by XPS and EPR analyses. EPR reveals the presence of two distinct types of oxygen vacancies: Type-A of V_o_ clusters and type-B of V_o_ monomers. Our results reveal that the presence of surface-localized type-B oxygen vacancies greatly enhances the photocatalytic H_2_ evolution production by efficient charge-carrier separation as well as suppressed electron–hole recombination. M–S and EIS studies further validated these findings with favorable shifts in the flat band potential, enhanced donor density, and decreased charge-transfer resistance in vacancy-rich materials. OCP decay measurements further confirmed the prolonged photogenerated carrier lifetimes in Type-B enriched STO (A-STO#2). Interestingly, the material prepared under the highest flame enthalpy conditions (A-STO#2) exhibited a 2-fold increase in H_2_ production relative to pristine STO perovskite, highlighting the beneficial role of engineered surface defects. These findings highlight the importance of defect chemistry in relation to the spin and lattice dynamics in perovskite oxide photocatalysts and shed new light on the design of vacancy/spin-controled highly efficient materials for solar-driven hydrogen evolution.

## Supplementary Material



## Notes

A-FSP process parameters used for the synthesis of SrTiO3-x nanoparticles; EIS parameters; O1 XPS spectra; EPR spectra; Raman spectra; and EPR analytical measurements of materials (PDF)
