# Supplementary material for: Interactions of Oxygen Vacancies with Photoinduced {Hole/Electron} Pairs in SrTiO3–x : Their Key Role in Photocatalytic H2 Production
Source: J Phys Chem C Nanomater Interfaces. 2025 Aug 15;129(34):15309–21. doi: 10.1021/acs.jpcc.5c03464 (PMC12403159; doi:10.1021/acs.jpcc.5c03464)

Supporting Information for Publication

Interactions of Oxygen-Vacancies with photoinduced {hole/electron} pairs in $SrTiO_{3-x}$: their key-role in photocatalytic H_2-production

*Areti Zindrou[1], Loukas Belles[1] and Yiannis Deligiannakis[1, *]*

[1]Laboratory of Physical Chemistry of Materials & Environment, Department of Physics, University of Ioannina, GR-45110 Ioannina, Greece

* Corresponding author: Yiannis Deligiannakis (ideligia@uoi.gr)

Contents

Supplementary Tables 2

Table S1: A-FSP process parameters used for the synthesis of $SrTiO_{3-x}$ nanoparticles....................2

Table S2: Electrochemical Impedance Spectroscopy (EIS) parameters....................2

Supplementary Figures....................3

Figure S1: O1s XPS spectra of material STO, R-STO#1 and A-STO#1. The dotted lines indicate shifts in the lattice oxygen and oxygen vacancy-related peaks. 3

Figure S2: EPR spectra recorded at 77K for (a) material A-STO#1 and (b) material R-STO#1. The solid line represents the experimental spectra while the dotted lines represent the simulated spectra using Easyspin MATLAB toolbox....................4

Figure S3: Raman spectra from 900 cm^{-1} up to 1700 cm^{-1} showcasing the absence of carbon Raman bands at 1350 cm^{-1} and 1590 cm^{-1} for D- and G- band respectively....................5

Figure S4: EPR analytical measurements of materials (a) STO, (b) A-STO#2 and (c) R-STO#2 used for the power saturation plots. 6

Supplementary Tables

Table S1: A-FSP process parameters used for the synthesis of $SrTiO_{3-x}$ nanoparticles

Material	Pilot Flame O_2/CH_4 ($L\cdot min^{-1}$)	P/D	Sheath Gas ($L\cdot min^{-1}$)	CH_4 inflow ($L\cdot min^{-1}$)
STO	4/2	5/5	O_2: 10	0
A-STO#1			N_2: 5	Axial CH_4: 1
A-STO#2			N_2: 5	Axial CH_4: 2
R-STO#1			N_2: 10	Radial CH_4: 3
R-STO#2			N_2: 10	Radial CH_4: 5

Table S2: Electrochemical Impedance Spectroscopy (EIS) parameters

	Electrodes	Rs (Ω)	CPE-T (× 10^{-6} F)	CPE-P	R_p (× 10^{6} Ω)
Dark	STO	605 ± 1	8.574 ± 0.007	0.969 ± 0.001	15 ± 2
	A-STO#2	598 ± 1	7.477 ± 0.004	0.967 ± 0.001	5.9 ± 0.1
	R-STO#2	600± 1	8.311± 0.002	0.964 ± 0.006	6.7 ± 0.2
Light	STO	629 ± 1	11.020 ± 0.004	0.946 ± 0.009	0.25 ± 0.03
	A-STO#2	608 ± 2	9.399 ± 0.0 05	0.949 ± 0.002	0.15 ± 0.02
	R-STO#2	619 ± 2	1.212 ± 0.004	0.950 ± 0.002	0.21 ± 0.01

Supplementary Figures

Figure S1: O1s XPS spectra of material STO, R-STO#1 and A-STO#1. The dotted lines indicate shifts in the lattice oxygen and oxygen vacancy-related peaks.

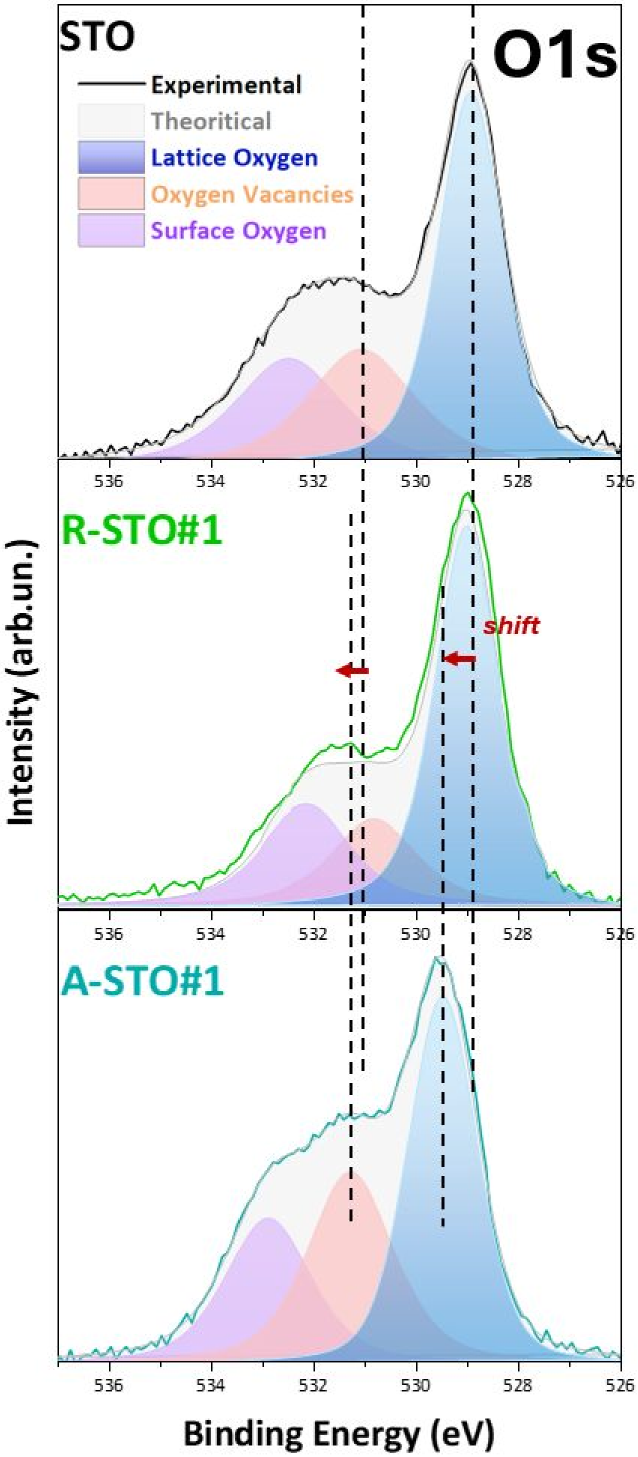

Figure S2: EPR spectra recorded at 77K for (a) material A-STO#1 and (b) material R-STO#1. The solid line represents the experimental spectra while the dotted lines represent the simulated spectra using Easyspin MATLAB toolbox.

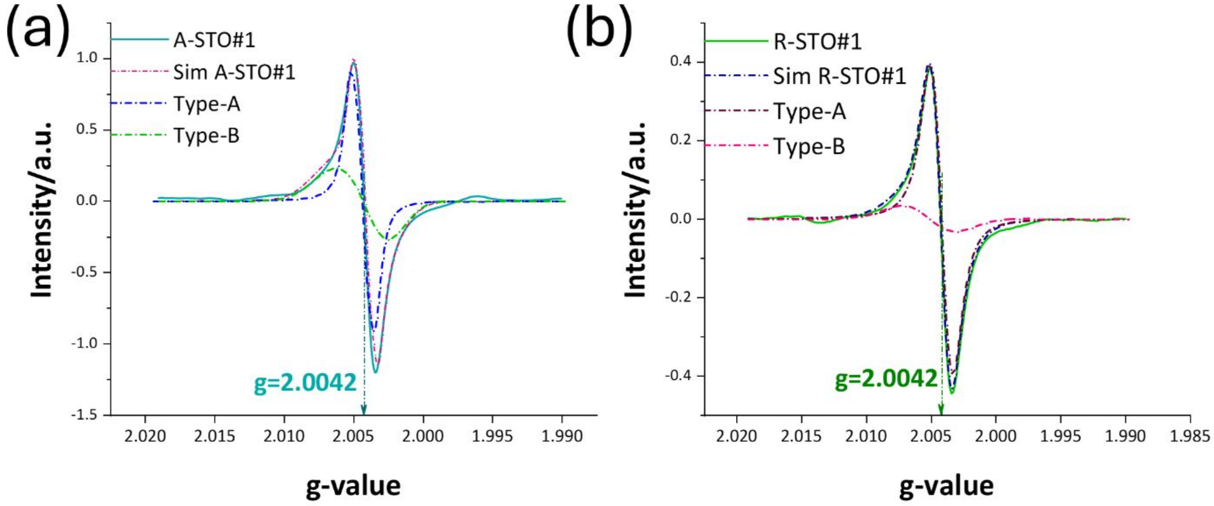

Figure S3: Raman spectra from 900 cm^{-1} up to 1700 cm^{-1} showcasing the absence of carbon Raman bands at 1350 cm^{-1} and 1590 cm^{-1} for D- and G- band respectively

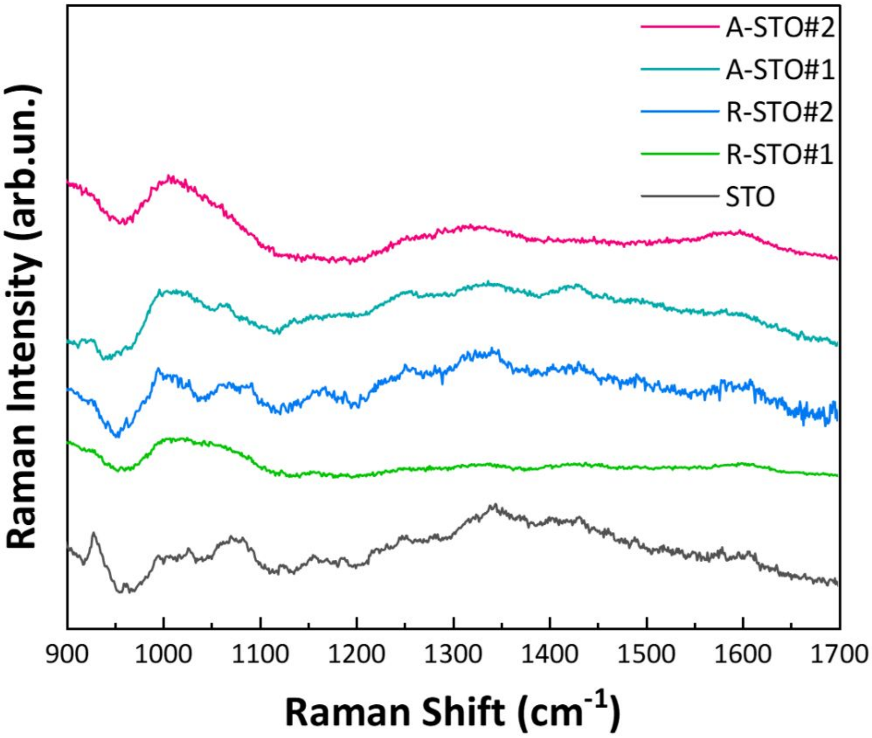

Figure S4: EPR analytical measurements of materials (a) STO, (b) A-STO#2 and (c) R-STO#2 used for the power saturation plots.

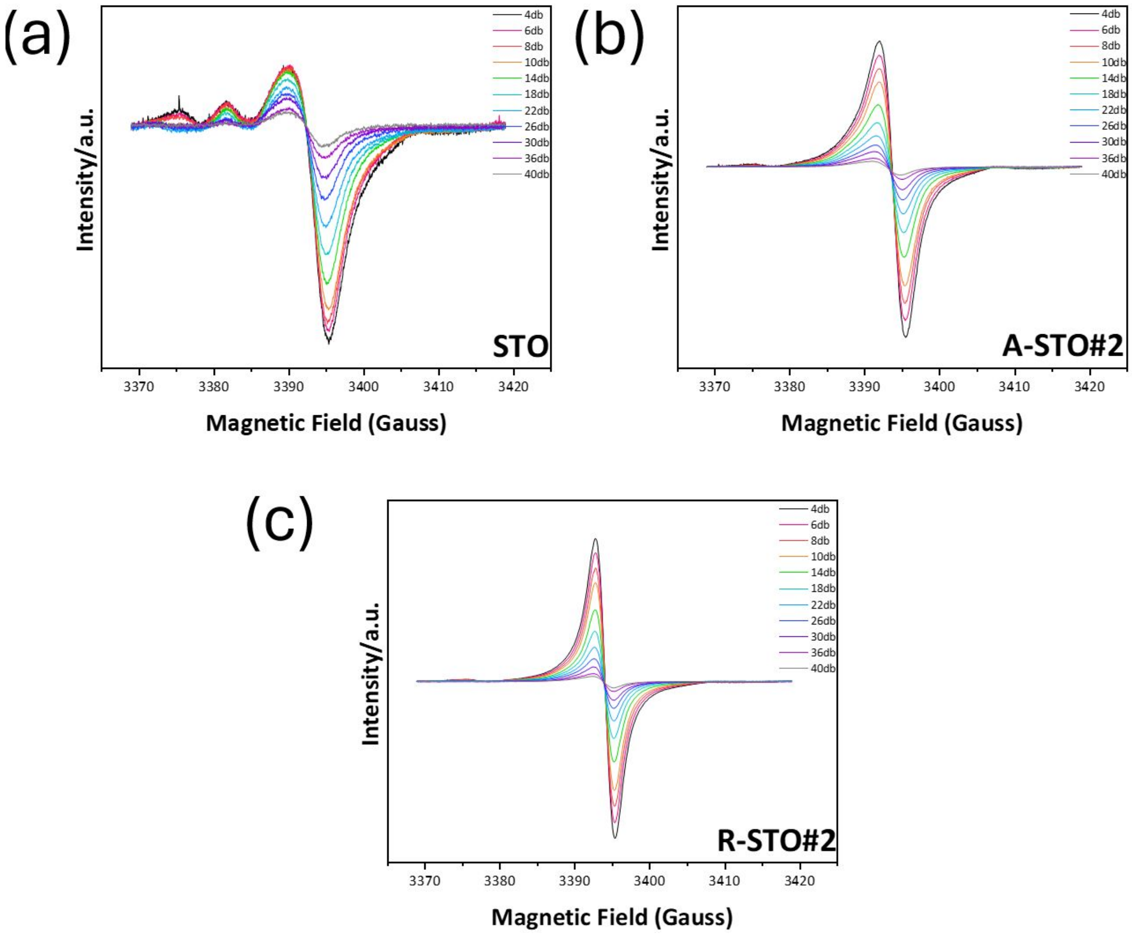

Supplement: Supplementary file 1 [file jp5c03464_si_001.pdf]
